# Adoption of the Citation Typing Ontology by the *Journal of Cheminformatics*

**DOI:** 10.1186/s13321-020-00448-1

**Published:** 2020-07-28

**Authors:** Egon Willighagen

**Affiliations:** Dept of Bioinformatics-BiGCaT, NUTRIM, Maastricht University, Universiteitssingel 50, 6229 ER Maastricht, The Netherlands

As authors, we cite literature for many reasons. The reasons are normally positive: it supports a statement we make in our article, the new work extends earlier ideas, or the cited paper outlines a method or a dataset we use. Sometimes, however, we cite an article differently, such as when we disagree with the conclusions from that article. Citations help us find more information about a concept and allow individual journal article to focus on the new content. Furthermore, they position the new work in its historical context and citation analyses can point us to research topics we would otherwise not have thought of [1].

Of course, citations have found additional uses that stem from the idea that articles that are cited a lot may be important. If we assume that all citations to an article are positive, this is a logical conclusion. However, citations are not always positive. We can cite an article because we disagree with the statements. For example, a 2011 paper in Science about the possible inclusion of arsenate ions in DNA has seen mostly disagreeing citations [2]. Then the article is important for a different reason.

This was picked up 10 years ago, when Shotton et al. published an ontology that formalizes a hierarchy of reasons: the Citation Typing Ontology (CiTO, purl.org/spar/cito) [3]. This ontology defines a citation as the act of citing some article. That allows one to make statements about the citation, in a machine readable way. Using the CiTO we can say the citation is neutral (*cito:citesAsAuthority*), positive (*cito:confirms*), or negative (*cito:disagreesWith*). The ontology also allows us to indicate reuse of methods and software (*cito:usesMethodIn*) and data (*cito:usesDataFrom*). This, of course, is closely related to recent efforts in data citation [4] and software citation [5]. The adoption of the CiTO, however, has so far not been wide in publishing. CiteULike [6] was one of the first tools that had support [7]. It allowed users to create citations with CiTO typing (see Fig. 1).

**Fig. 1:**
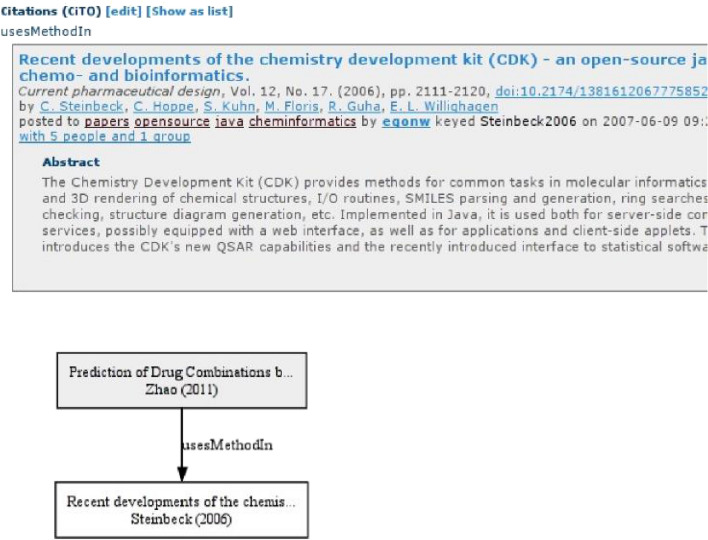
Screenshot of CiteUlike showing the citation between two articles and that the reason is that the citing article uses the method proposed in the cite article (*cito:usesMethodIn*)

## Adopting the CiTO

If the past 10 years has shown anything, it is that the activity of scholarly communication via journal articles is not easily changed. Whether it is widespread adoption of data repository, minimal reporting standards, or freely sharing citations, the interest may be there, but the uptake is slow. The OpenCitations project [8, 9] and Initiative for Open Citations [10] show how hard it is to change the momentum. And while CiteULike introduced support for the CiTO, other references managers have not (yet). A chicken-and-egg situation may be an underlying issue: if there are no providers of CiTO annotation, why should tools that work with citations use it? And at the same time, if there is no use of it, why invest effort to provide such annotation.

However, the *Journal of Cheminformatics* considers adoption important. For example, we may want to learn what articles are using a method proposed in some article. We may want to see how data is reused, or we may want to get warned that we are citing an article that has been refuted repeatedly.

Therefore, we are starting a pilot to roll out CiTO annotation in the *Journal of Cheminformatics*. We take advantage here of the ability to add notes to *full form* (see [3]) references in bibliographies. These are referred to as *bibnotes*. The content of the note will be strictly formatted: it will use the syntax *[cito:usesMethodIn]* and formatted in bold. That is, the bibnote starts with the *[* character, followed by one of the CiTO types, and ending with the* ]* character. If you wish to provide more than one annotation, you can repeat this syntax, separated by one or more spaces, for example: *[cito:usesMethodIn][cito:citeAsAuthority]*. By using this specific syntax, we introduce a level of machine readability such that this annotation can be extracted with text mining approaches and used by downstream citation projects.

These bibnotes can be used to overwrite the default *cito:cites*. We currently encourage authors interested in participating in this pilot to use the following CiTO types: *cito:citesAsDataSource* when you use data in your paper from the cited source, *cito:usesMethodIn* when you use a method from the cites source, *cito:citesAsAuthority* for articles that you cite as authorative works in the field, *cito:discusses* when you discuss the content of the cited article, and *cito:extends* when your article describes a new release of software or database described in the cited article. However, you are free to use any of the other CiTO types, including *cito:agreesWith* and *cito:disagreesWith*.

We also plan to adopt this approach for comments (*cito:repliesTo*) and errata/corrigenda/corrections (*cito:updates*). These annotations will be handled at an editorial level.

With this pilot we hope to trigger further adoption of approaches like CiTO. We plan to use this information in WikiCite [11] and Scholia [12] to demonstrate downstream use, but hope that projects like OpenCitations and SciGraph (www.springernature.com/gp/researchers/scigraph) will pick it up too. During the pilot, we will also develop practical guidance on how to use reference managers and type setting tools like Microsoft Word and LaTeX can be used to add these annotations.

Let this be the egg or chicken (depending on your philosophy), we are looking to innovate how we cite our literature.
